# Transmissibility and Disease Progression of Asymptomatic *Mycobacterium* *tuberculosis* Infection, Lima, Peru

**DOI:** 10.3201/eid3204.251947

**Published:** 2026-04

**Authors:** Ruitong Wang, Chuan-Chin Huang, Mercedes C. Becerra, Roger I. Calderon, Carmen C. Contreras, Jerome T. Galea, Judith Jimenez, Leonid Lecca, Rosa M. Yataco, Zibiao Zhang, Megan B. Murray

**Affiliations:** Harvard T.H. Chan School of Public Health, Boston, Massachusetts, USA (R. Wang, M.B. Murray); Harvard Medical School, Boston (C.-C. Huang, M.C. Becerra, L. Lecca, M.B. Murray); Brigham and Women’s Hospital, Boston (C.-C. Huang, Z. Zhang, M.B. Murray); Advanced Research and Health, Lima, Peru (R.I. Calderon); Universidad de San Martin de Porres, Lima (R.I. Calderon); Partners In Health—Socios En Salud Sucursal Peru, Lima (C.C. Contreras, J. Jimenez, L. Lecca, R.M. Yataco); University of South Florida, Tampa, Florida, USA (J.T. Galea)

**Keywords:** tuberculosis and other mycobacteria, bacteria, Mycobacterium tuberculosis, asymptomatic diseases, disease transmission, respiratory infections, Peru

## Abstract

Estimating the transmissibility of asymptomatic *Mycobacterium tuberculosis* infection can clarify its contribution to tuberculosis (TB) spread. We conducted a prospective cohort study in Lima, Peru, enrolling index TB patients and their household contacts (HHCs) and classifying patients by the presence of symptoms including cough, night sweats, weight loss, or fever. We followed HHCs with serial tuberculin skin testing and clinical evaluations. Among 4,296 child HHCs, adjusted estimates for baseline infection (prevalence ratio 0.62 [95% CI 0.37–1.03]), incident infection at 6 months (hazard ratio (aHR) 0.63 [95% CI 0.27–1.49]), and TB disease during 1 year of follow-up (aHR 0.74 [95% CI 0.35–1.56]) were all consistent with lower risk for infection and disease progression among HHCs of asymptomatic compared with symptomatic index patients. Although asymptomatic infections may be less transmissible than symptomatic infections, the high prevalence of asymptomatic patients in national surveys suggest that they may contribute substantially to transmission.

Tuberculosis (TB) remains a global health concern, with an estimated 10.8 million persons falling ill in 2024 (*1*). National TB prevalence surveys show that a large proportion of persons with bacteriologically confirmed *Mycobacterium tuberculosis* infection do not report symptoms during screening (*2*). This group is considered to have subclinical TB, which the World Health Organization (WHO) terms asymptomatic TB (*3*). Traditional TB control strategies focus on symptomatic persons seeking care at healthcare facilities or on screening algorithms that initiate testing among those who report symptoms (*4*). Asymptomatic patients and those with mild or nonspecific symptoms that are not recognized during symptom-based screening (i.e., minimally symptomatic TB) (*5*) are often missed and may contribute to transmission.

Estimating the transmissibility of asymptomatic TB may clarify its contribution to TB transmission. Although studies suggest that asymptomatic TB can be transmitted (*6*,*7*), it remains unclear how its infectiousness and potential for disease progression compares with symptomatic TB. Most evidence comes from national prevalence surveys that compared infection prevalence among household contacts (HHCs) of symptomatic and asymptomatic index patients but did not assess incident infection or incident disease (*8*,*9*).

Using data from a longitudinal cohort of TB index patients and their HHCs in Lima, Peru, we assessed the relative transmissibility of TB and risk for disease progression after exposure to TB patients who did not report symptoms when they tested positive for *M. tuberculosis*. We compared infection prevalence at enrollment among HHCs exposed to asymptomatic versus symptomatic index patients as a proxy for baseline transmission and evaluated incident infection at 6 and 12 months to assess ongoing transmission risk. We then compared 12-month disease incidence to estimate progression potential and examined transmission risk factors among asymptomatic index patients. Together, those analyses provide empirical estimates of the transmissibility and disease progression potential of asymptomatic TB and clarify its contribution to overall TB transmission.

## Materials and Methods

### Study Design and Participants

We conducted a prospective cohort study among HHCs of index TB patients in Lima, Peru, during September 2009–September 2012. We recruited patients >15 years of age with newly diagnosed pulmonary TB from 106 district health centers in Lima. TB diagnosis required either microbiological evidence (positive sputum smear or culture) or a clinician’s judgment based on chest radiograph, clinical manifestation, or both. We collected demographic and clinical data for index patients, including age, sex, employment status, symptom status, HIV status, smoking, alcohol use, diabetes, socioeconomic status, and sputum smear results.

Within 2 weeks of index patient enrollment, we identified and enrolled HHCs and collected data on age, sex, HIV status, smoking, alcohol use, diabetes, bacille Calmette–Guérin (BCG) vaccination status, and body mass index (BMI). We assessed baseline *M. tuberculosis* infection using the tuberculin skin test (TST) among HHCs without a prior positive TST or TB disease. We considered a TST positive at >10 mm induration in HIV-uninfected persons and >5 mm in those with HIV. We retested HHCs with a prior negative TST at 6 and 12 months. We followed up with HHCs at 2, 6, and 12 months to assess TB symptoms and document interval diagnoses; we referred symptomatic participants for clinical evaluation and reviewed medical records to identify TB diagnoses during follow-up. Additional study design details have been published elsewhere (*10*).

Ethics committees at Harvard University and at the National Institute of Health in Peru approved the study. All study participants or their guardians provided written informed consent, and children <18 years old provided assent.

### Exposure and Outcomes

We stratified index patients by the presence of baseline symptoms included in the WHO 4-symptom screen (W4SS) (*11**,**12*): cough, night sweats, weight loss, and fever. We evaluated 4 outcomes: baseline TB infection among HHCs; incident infection over 6 months among those uninfected at baseline; incident infection over 12 months among those uninfected at baseline; and incident TB disease over 12 months among HHCs without coprevalent TB at baseline. 

We classified HHCs as infected at baseline if they had TB disease or a positive TST at enrollment. To reduce misclassification of community-acquired infection as household transmission, we excluded HHCs with a prior history of TB disease or a positive TST from the analysis. We considered HHCs to have incident TB infection if they were uninfected at baseline and subsequently had a positive TST or experienced secondary TB disease. We considered HHCs to have incident TB disease if they received a diagnosis of TB disease at any time from 2 weeks after enrollment to the end of follow-up.

### Statistical Analysis

We included only index patients with microbiologically confirmed TB. To assess the association between index patient symptom status and baseline HHC infection, we used generalized estimating equations with modified Poisson regression, specifying an exchangeable correlation structure to account for clustering within households. We estimated prevalence ratios (PR) and 95% CIs. We evaluated associations with incident TB infection at 6 and 12 months and incident TB disease at 12 months using Cox frailty models and reported hazard ratio (HRs) and 95% CIs.

We first fitted univariable models, then multivariable models, adjusting for prespecified confounders and predictors of TB infection or disease. Covariates included index patient age group, HIV status, smoking, alcohol use, diabetes, and household socioeconomic status. Additional predictors of HHC infection included index patient sex and employment status and HHC sex, age group, HIV status, diabetes status, BCG vaccination status, smoking and alcohol use, and BMI category (Appendix Table 1). A second multivariable model retained confounders and variables with p<0.10 in the initial multivariable analysis. Because sputum smear status might lie on the causal pathway between disease severity and transmission, we did not include it in regression models.

For analyses of TB infection, we restricted the primary sample to child HHCs <15 years of age because infection in that group is more consistent with recent household transmission. Sensitivity analysis included HHCs of all ages. Analyses of incident TB disease included HHCs of all ages, given the relative rarity of disease compared with infection. We conducted complete-case analyses, excluding 13% of observations (Appendix Table 2), and assessed robustness using multiple imputation. Finally, we examined specific symptom patterns by classifying index patients into 4 groups: asymptomatic; cough only; no cough but >1 of night sweats, weight loss, or fever; and cough plus >1 of those symptoms. We evaluated associations between symptom patterns and baseline infection and 6-month incident infection among child HHCs.

We next explored factors associated with transmission or disease progression from asymptomatic index patients. For that exploratory analysis, we restricted the sample to HHCs of asymptomatic index cases. We estimated PRs for baseline TB infection and odds ratios (ORs) for incident TB infection at 6 months and incident TB disease at 12 months, to identify potential associated factors. Given the limited sample size in the subgroup, we limited analyses to univariable models examining associations between characteristics of asymptomatic index patients and each outcome among their HHCs.

## Results

We identified 3,109 microbiologically confirmed *M. tuberculosis*–infected index patients with known baseline symptom status (Appendix Table 3), including 113 asymptomatic and 2,996 symptomatic patients. Asymptomatic patients were significantly more likely to be smear-negative (OR 3.15 [95% CI 2.14–4.65]; p<0.001).

We enrolled 12,230 HHCs (Appendix Table 4), of whom 4,296 (35.1%) were <15 years of age (Table 1). Most (80.9%) had a BCG scar; 62.4% were <30 years of age, 57.8% had normal BMI, 6.0% were current smokers, and 25.7% were current drinkers.

**Table 1:** Characteristics of household contacts <15 years of age by symptom status of tuberculosis index patients in study of transmissibility of asymptomatic *Mycobacterium tuberculosis* infection, Lima, Peru*

Variable	No. (%) contacts		
	Total	Symptomatic index patient	Asymptomatic index patient
Age group, y, n = 4,296			
0–4	1,558 (36.3)	1,502 (36.1)	56 (42.4)
5–9	1,312 (30.5)	1,272 (30.5)	40 (30.3)
10–15	1,426 (33.2)	1,390 (33.4)	36 (27.3)
Sex, n = 4,296			
M	2,158 (50.2)	2,091 (50.2)	67 (50.8)
F	2,138 (49.8)	2,073 (49.8)	65 (49.2)
HIV status, n = 4,247			
Negative	4,243 (99.9)	4,115 (99.9)	128 (100)
Positive	4 (0.1)	4 (0.1)	0
BCG scar, n = 4,296			
No	820 (19.1)	801 (19.2)	19 (14.4)
Yes	3,476 (80.9)	3,363 (80.8)	113 (85.6)
Diabetes, n = 4,283			
No	4,281 (99.9)	4,149 (99.9)	132 (100)
Yes	2 (0.1)	2 (0.1)	0
Smoking, n = 4,294			
Nonsmoker	4,288 (99.9)	4,156 (99.9)	132 (100)
Smoker	6 (0.1)	6 (0.1)	0
Alcohol consumption, n = 4,293			
Nondrinker	4,257 (99.2)	4,126 (99.2)	131 (99.2)
Drinker	36 (0.8)	35 (0.8)	1 (0.8)
BMI category, n = 4,253†			
Normal	3,420 (80.4)	3,316 (80.4)	104 (79.4)
Underweight	116 (2.7)	109 (2.6)	7 (5.3)
Overweight	717 (16.9)	697 (16.9)	20 (15.3)

*BCG, bacille Calmette–Guérin; BMI, body mass index. †For participants >19 years of age, we defined underweight as BMI <18.5 kg/m^2^, normal as 18.5–24.9 kg/m^2^, and overweight as BMI >25 kg/m^2^. For participants <19 y, we defined status using World Health Organization BMI-for-age z-scores: underweight z-score <–2, normal z-score –2 to 2, and overweight z-score >2.

### Baseline Infection

Among child HHCs, 23.0% exposed to symptomatic index patients and 15.0% exposed to asymptomatic index patients were TST-positive at baseline (Table 2). Exposure to asymptomatic index patients was associated with lower baseline infection (crude PR 0.60 [95% CI 0.36–1.01]). Adjusted PR was 0.62 (95% CI 0.37–1.03) for model A, in which we adjusted for age, sex, HIV status, alcohol consumption status, BCG vaccination, and BMI category, and 0.61 (95% CI 0.36–1.03) in model B, in which we excluded sex and alcohol consumption from the adjusted variables. When all HHCs were included, the association was attenuated (adjusted PR 0.94 [95% CI 0.79–1.11]) (Appendix Table 5). Multiple imputation yielded similar estimates, with lower prevalence among child HHCs (adjusted PR 0.58 [95% CI 0.35–0.95]) and comparable results among all HHCs (adjusted PR 0.88 [95% CI 0.74–1.04]) (Appendix Table 6).

**Table 2:** Risk for *Mycobacterium tuberculosis* infection at baseline among household contacts of tuberculosis index patients in study of transmissibility of asymptomatic *M.* *tuberculosis* infection, Lima, Peru*

Symptom status	No. contacts	No. (%) baseline infection	Univariate model			Multivariate model A†			Multivariate model B‡	
			Crude PR (95% CI)	p value		Adjusted PR (95% CI)	p value		Adjusted PR (95% CI)	p value
Symptomatic	3,586	824 (22.98)	Referent			Referent			Referent	
Asymptomatic	113	17 (15.04)	0.60 (0.36–1.01)	0.06		0.62 (0.37– 1.03)	0.07		0.61 (0.36–1.03)	0.06

*Household contacts are children <15 years of age. PR, prevalence ratio. †Multivariate model A was adjusted for the following characteristics of index patients: age, sex, HIV status, smoking status, alcohol consumption status, socioeconomic status, employment status, and diabetes; and the following characteristics of household contacts: age, sex, smoking status, alcohol consumption status, bacille Calmette–Guérin vaccination, and body mass index category. Diabetes and HIV status of household contacts were excluded due to sparse data in some of its categories, which led to unstable estimates and nonestimable coefficients in the model. ‡Multivariate model B did not adjust for employment status and sex of index patients.

### Incident Infection

At 6 months, 14.5% of child HHCs exposed to symptomatic index patients and 10.3% exposed to asymptomatic index patients tested positive by TST (Table 3). Exposure to asymptomatic index patients was associated with lower hazard of infection (crude HR 0.63 [95% CI 0.27–1.49], adjusted HR 0.62 [95% CI 0.26–1.51] in both multivariable models). Estimates were similar when all HHCs were included (adjusted HR 0.78 [95% CI 0.50–1.20]) (Appendix Table 7).

**Table 3:** Hazard ratios of *Mycobacterium tuberculosis* infection at 6-month follow-up among initially uninfected household contacts of tuberculosis patients in study of transmissibility of asymptomatic *M.* *tuberculosis* infection, Lima, Peru*

Symptom status	No. contacts	No. (%) incident infection	Univariate model			Multivariate model A†			Multivariate model B‡	
			Crude HR (95% CI)	p value		Adjusted HR (95% CI)	p value		Adjusted HR (95% CI)	p value
Symptomatic	2,204	320 (14.52)	Referent			Referent			Referent	
Asymptomatic	68	7 (10.29)	0.63 (0.27–1.49)	0.29		0.62 (0.26–1.51)	0.29		0.62 (0.26–1.51)	0.30

*Household contacts are children <15 years of age. HR, hazard ratio. †Multivariate model A was adjusted for the following characteristics of index patients: age, sex, HIV status, smoking status, alcohol consumption status, socioeconomic status, employment status, and diabetes; and the following characteristics of household contacts: age, sex, HIV status, alcohol consumption status, bacille Calmette–Guérin vaccination, and body mass index category. Diabetes and smoking status of household contacts excluded due to sparse data in some of its categories, which led to unstable hazard ratio estimates and nonestimable coefficients in the Cox model. ‡Multivariate model B did not adjust for sex and alcohol consumption of household contact.

At 12 months, infection occurred in 21.1% of child HHCs exposed to symptomatic index patients and 16.2% of those exposed to asymptomatic index patients, (Appendix Table 8). Hazard ratios remained lower for asymptomatic exposure among child HHCs (adjusted HR 0.73 [95% CI 0.35–1.50]) and all HHCs (adjusted HR 0.80 [95% CI 0.55–1.17]) (Appendix Table 9). Multiple imputation analyses were directionally consistent (Appendix Table 10).

### Incident Disease

Within 12 months, 3.0% of HHCs exposed to symptomatic index patients and 2.4% exposed to asymptomatic index patients experienced TB disease (Table 4). HRs again favored lower risk with asymptomatic exposure (crude HR 0.74 [95% CI 0.35–1.56]; adjusted HR 0.80 [95% CI 0.38–1.67] for model A, 0.81 [95% CI 0.39–1.69] for model B), with similar findings under multiple imputation (Appendix Table 10).

**Table 4:** Hazard ratios of incident tuberculosis disease among all household contacts of index patients by symptom status in study of transmissibility of asymptomatic *M.* *tuberculosis* infection, Lima, Peru*

Symptom status	No. contacts	No. (%) incident disease	Univariate model			Multivariate model A†			Multivariate model B	
			Crude HR (95% CI)	p value		Adjusted HR (95% CI)	p value		Adjusted HR (95% CI)	p value
Symptomatic	10,375	313 (3.02)	Referent			Referent			Referent	
Asymptomatic	410	10 (2.44)	0.74 (0.35–1.56)	0.43		0.80 (0.38–1.67)	0.55		0.81 (0.39–1.69)	0.57

* Household contacts are children <15 years of age. HR, hazard ratio. †Multivariate model A was adjusted for the following characteristics of index patients: age, sex, HIV status, smoking status, alcohol consumption status, socioeconomic status, employment status, and diabetes; and the following characteristics of household contacts: age, sex, HIV status, smoking status, alcohol consumption status, diabetes, bacille Calmette–Guérin vaccination, and body mass index category. ‡Multivariate model B did not adjust for employment status of index patient, and sex, smoking status, and alcohol consumption of household contacts

### Symptom Patterns and Infection Risk among Child HHCs

Baseline TST positivity among child HHCs was 15.0% for asymptomatic exposure, 12.4% for noncough symptoms only, 23.0% for cough only, and 24.0% for cough plus other symptoms (Table 5). Infection risk among children exposed to asymptomatic index patients was similar to risk among those exposed to index patients without cough but with other symptoms (adjusted PR 0.94–0.95 across models). At 6 months, infection risk was higher among children exposed to index patients with noncough symptoms only (adjusted HR 1.27–1.31), and larger still for cough only (adjusted HR 1.84) and cough plus other symptoms (adjusted HR 1.59–1.66) (Appendix Table 11).

**Table 5:** Risk for *Mycobacterium tuberculosis* infection at baseline among household contacts of tuberculosis index patients by symptom pattern in study of transmissibility of asymptomatic *M.* *tuberculosis* infection, Lima, Peru*

Symptom status†	No. contacts	No. (%) baseline infection	Univariate model			Multivariate model A‡			Multivariate model B§	
			Crude PR (95% CI)	p value		Adjusted PR (95% CI)	p value		Adjusted PR (95% CI)	p value
Asymptomatic	113	17 (15.04)	Referent			Referent			Referent	
Cough only	296	68 (22.97)	1.64 (0.92–2.90)	0.09		1.59 (0.90–2.80)	0.11		1.61 (0.92–2.82)	0.10
Noncough symptoms only	266	33 (12.41)	0.94 (0.51–1.73)	0.83		0.94 (0.51–1.72)	0.83		0.95 (0.52–1.75)	0.87
Cough and any noncough symptoms	2,964	712 (24.02)	1.73 (1.03–2.90)	0.04		1.67 (1.00–2.78)	0.05		1.68 (1.01–2.79)	0.05

*Household contacts are children <15 years of age. PR, prevalence ratio. †Noncough symptoms include fever, weight loss and night sweats. ‡Multivariate model A was adjusted for the following characteristics of index patients: age, sex, HIV status, smoking status, alcohol consumption status, socioeconomic status, employment status, and diabetes; and the following characteristics of household contacts: age, sex, smoking status, alcohol consumption status, bacille Calmette–Guérin vaccination, and body mass index category. Diabetes and HIV status of household contacts excluded due to sparse data in some of its categories, which led to unstable estimates and nonestimable coefficients in the model. §Multivariate model B did not adjust for employment status of index patients and sex, bacille Calmette–Guérin scar, alcohol consumption, and body mass index category of household contacts.

### Cough Duration and Infection Risk

Among child HHCs, longer cough duration in symptomatic index patients was associated with progressively higher baseline infection compared with asymptomatic exposure. Adjusted PRs increased as cough duration increased (Appendix Table 13). PR was 1.28 (95% CI 0.74–2.19) for 0–13 days up to PR 1.76 (95% CI 1.04–2.98) for >56 days, consistent with a dose-response pattern. Adjusted hazard ratios for incident infection and disease similarly increased with longer cough duration.

### Transmission Potential among Asymptomatic Index Patients

Among asymptomatic index patients, HIV infection was associated with lower baseline infection among HHCs (PR 0.54 [95% CI 0.32–0.92]) (Appendix Table 12). At 6-month follow-up, we observed higher odds of infection among HHCs exposed to asymptomatic patients 46–60 years of age (OR 3.00 [95% CI 1.41–6.37]) and with high socioeconomic status (OR 4.30 [95% CI 1.49–12.40]). We observed lower odds among HHCs of asymptomatic patients with HIV (OR 0.37 [95% CI 0.19–0.74]) and those >61 years of age (OR 0.40 [95% CI 0.19–0.89]) (Table 6). We observed no significant associations for incident disease (Appendix Table 14).

**Table 6:** Association between characteristics of asymptomatic index patients and risk for *Mycobacterium tuberculosis* infection at 6-month follow-up of all household contacts in study of transmissibility of asymptomatic *M.* *tuberculosis*, Lima, Peru*

Characteristic	No. (%) uninfected contacts	No. (%) infected contacts	Odds ratio (95%CI)	p value
Age group, y, n = 230				
16–30	92 (50.5)	26 (54.2)	Referent	Referent
31–45	20 (11.0)	6 (12.5)	1.08 (0.44–2.68)	0.87
46–60	12 (6.59)	10 (20.8)	3.00 (1.41–6.37)	0.004
≥61	58 (31.9)	6 (12.5)	0.40 (0.19–0.89)	0.02
Sex, n = 230				
M	143 (78.6)	42 (87.5)	Referent	Referent
F	39 (21.4)	6 (12.5)	0.48 (0.17–1.37)	0.17
HIV status, n = 230				
Negative	131 (72.0)	42 (87.5)	Referent	Referent
Positive	51 (28.0)	6 (12.5)	0.37 (0.19–0.74)	0.005
Smoking status, n = 230				
Nonsmoker	171 (94.5)	45 (93.8)	Referent	Referent
Smoker	10 (5.52)	3 (6.25)	1.02 (0.40–2.60)	0.85
SES, n = 222†				
Low	58 (32.6)	10 (22.7)	Referent	Referent
Medium	105 (59.0)	23 (52.3)	1.36 (0.61–3.03)	0.46
High	15 (8.43)	11 (25.0)	4.30 (1.49–12.4)	0.007
Employment status, n = 228				
Stay at home	121 (67.2)	37 (77.1)	Referent	Referent
Work outside	59 (32.8)	11 (22.9)	0.54 (0.26–1.14)	0.11
Alcohol consumption, n = 208				
Drinker	41 (24.8)	17 (39.5)	Referent	Referent
Nondrinker	124 (75.2)	26 (60.5)	0.53 (0.27–1.03)	0.06
Diabetes, n = 230				
No	179 (98.4)	47 (97.9)	Referent	Referent
Yes	3 (1.65)	1 (2.08)	1.21 (0.12–11.9)	0.87

*Results of univariate analysis. SES, socioeconomic status. †We created a continuous variable to capture summarize household-level socioeconomic status by including variables on housing quality, water supply and sanitation in a principal component analysis. The continuous SES score was categorized into tertiles corresponding to relative low, middle, and upper SES.

## Discussion

In this study, baseline and incident infection among HHCs were lower after exposure to asymptomatic versus symptomatic index patients. Incident disease estimates were also lower, although 95% CIs were wide and included the null (no effect). Because we recruited index patients from local clinics, most were experiencing symptoms when they sought care, limiting the number of asymptomatic cases. Given the high prevalence of asymptomatic TB reported in national surveys, our findings are consistent with the possibility that asymptomatic TB contributes meaningfully to transmission, although the magnitude remains uncertain.

Our infection estimates are broadly consistent with those from previous studies. A 2007 survey in Vietnam found that child HHCs exposed to symptomatic index patients had more than twice the risk for TST positivity compared with those exposed to asymptomatic patients (*9*). In Brazil, a prospective cohort reported 1.5-fold higher odds of TST conversion among contacts of patients with prolonged cough (>4 weeks) (*13*). Those effects were somewhat stronger than ours, possibly reflecting differences in infection definitions (e.g., TST cutoffs) and symptom classification. A pooled analysis from Vietnam, Bangladesh, and the Philippines reported an OR of 1.2 for infection (*8*), closely aligning with our converted estimate. Those studies were conducted in settings dominated by lineages 1 and 2, whereas lineage 4 predominates in Peru (*14*). Because *M. tuberculosis* lineages differ in host immune response, disease severity, and transmissibility (*15*), lineage distribution might influence relative transmission from asymptomatic versus symptomatic patients. Molecular epidemiologic data also support transmission from asymptomatic patients; whole-genome sequencing and phylogenetic modeling suggested that 514 patients transmitted TB before symptom onset (*16*).

Several mechanisms may explain transmission from asymptomatic patients. First, aerosolization of *M. tuberculosis* bacilli is not limited to coughing. Exhaled *M. tuberculosis* has been detected in the absence of cough (*6*,*17*); 1 study estimated that tidal breathing accounted for 93% of daily aerosolized *M. tuberculosis* (*7*), although participants were symptomatic. Second, some patients classified as asymptomatic may experience nonspecific symptoms they consider normal and therefore do not report. In Lima, chronic cough related to air pollution may reduce reporting of symptom changes; in 2011–2014, average concentration of particulate matter with a diameter <2.5 μm (PM_2.5_) levels were approximately twice the then-current WHO annual guideline of 10 μg/m^3^ (*18*). Such misattribution could result in underreporting and misclassification of symptomatic patients as asymptomatic, inflating observed transmission risk. Similar misclassification could arise from smoking-related cough or seasonal respiratory symptoms (*19*,*20*).

We classified TB patients on the basis of self-reported symptoms at the time patients sought care, a process shaped by individual interpretation, stigma, timing of assessment, and interview practices (*21*). Although widely used in clinical settings, dichotomizing symptom status is increasingly recognized as imprecise and lacking clear biologic validity as a marker of disease severity (*21*,*22*).

Although they are not designed to estimate population-level contribution of asymptomatic TB, national prevalence surveys and meta-analyses consistently report that 50%–60% of bacteriologically confirmed cases are asymptomatic; some analyses suggested even higher proportions after accounting for diagnostic misclassification (*2*,*23*–*25*). If a conservative estimate of 60% asymptomatic disease is considered alongside our adjusted hazard ratio of 0.6 for incident infection among child HHCs, and if that hazard ratio reflects proportional differences in per-unit-time infectiousness, then asymptomatic patients could account for a substantial share of transmission, potentially approaching one half. That interpretation assumes comparable exposure duration and minimal unmeasured confounding and should be viewed as an illustrative extrapolation rather than a formal transmission model.

Our estimates align with modeling studies attributing a large fraction of TB transmission to asymptomatic or subclinical disease. One analysis of 14 high-burden countries estimated that 68% of transmission arose from asymptomatic cases (*6*), and another projected that subclinical patients accounted for 50%–62% of transmission over 5 years across multiple countries in Asia (*26*). Those findings consistently suggest the limits of symptom-based screening. Passive case finding, or algorithms that trigger testing only after symptoms appear, inevitably miss large numbers of infectious persons. By quantifying the transmissibility of asymptomatic patients, our study adds to evidence that symptom-independent approaches are needed to improve early detection, reduce transmission, and strengthen TB control.

A limitation of our study is that the small number of asymptomatic TB patients in our sample limits our analysis power and constrains the strength of inference. Second, we cannot rule out the possibility of symptom status misclassification from underreporting. Third, within the high-transmission setting in Lima, some HHCs may have acquired TB infection in the community rather than the household. Such misclassification of the source of infection is likely nondifferential to the symptom status of index patients, which would bias the relative transmissibility of asymptomatic index patients toward the null result of no effect. To address that possibility, we focused on child HHCs, for whom recent household transmission data are more consistent. We excluded participants with a history of positive TST or TB disease; baseline TST positivity could be influenced by cumulative exposure and is therefore an imperfect proxy for transmissibility from index patients. We were not able to directly measure the duration of exposure between HHCs and index patients. If asymptomatic TB patients received diagnosis earlier in the course of disease (*27*), their HHCs may have experienced a shorter period of exposure before diagnosis. Our finding that longer reported coughing duration was associated with higher risks of infection and disease among HHCs underscores the importance of cumulative exposure over time. Those results support the interpretation that part of the lower risk observed among contacts of asymptomatic patients reflects reduced cumulative infectious exposure rather than differences in intrinsic infectiousness per unit time. At the same time, because symptom duration is a proxy for total exposure and we were not able to directly incorporate exposure time into our primary models, we cannot exclude the possibility that asymptomatic TB may also be less infectious per unit time than symptomatic disease. Our findings, therefore, reflect differences in cumulative transmission risk; uncertainty remains about the contribution of biologic infectiousness per unit time. Furthermore, prior BCG vaccination might contribute to the false-positive TST reactions, particularly in young children (*28*). In Peru, BCG vaccination is administered at birth; evidence suggests that vaccination in infancy has limited effect on TST interpretation (*29*). We used a cutoff of 10 mm, even for children <5 years of age; we adjusted for BCG vaccination status in all models to reduce potential confounding. Last, because our study was a large prospective cohort in Peru, the ability to generalize our results is limited, particularly to settings with low TB incidence and different dominant lineages.

In conclusion, our findings are consistent with lower infectiousness and lower risk for disease progression among HHCs exposed to asymptomatic TB patients than for those exposed to symptomatic patients. Given the high prevalence of asymptomatic TB reported in national surveys, our results highlight the limitations of symptom-triggered testing within current TB screening algorithms and are consistent with the potential value of symptom-independent approaches to improve early detection and reduce transmission.

Appendix Additional information about transmissibility and disease progression of asymptomatic *Mycobacterium tuberculosis,* Lima, Peru.
