# Supplementary material for: Transmissibility and Disease Progression of Asymptomatic Mycobacterium tuberculosis Infection, Lima, Peru
Source: Emerg Infect Dis. 2026 Apr;32(4):584–91. doi: 10.3201/eid3204.251947 (PMC13094839; doi:10.3201/eid3204.251947)
Supplement: Appendix — Additional information about transmissibility and disease progression of asymptomatic Mycobacterium tuberculosis, Lima, Peru. [file 25-1947-Techapp-s1.pdf]

Article DOI: https://doi.org/10.3201/eid3104.251947

EID cannot ensure accessibility for supplementary materials supplied by authors. Readers who have difficulty accessing supplementary content should contact the authors for assistance.

Transmissibility and Disease Progression of Asymptomatic *Mycobacterium tuberculosis* Infection, Lima, Peru

Appendix

Appendix Table 1. Definition of variables included in the analysis

Variables	Definition	Reference category	Variable type
Confounders (index patients characteristics)			
Age group	16–30;<br>31–45;<br>46–60;<br>61 and older;	16 - 30	Categorical
HIV status	HIV status from lab test and self-report	Negative	Binary
Smoking	Smoking status: non-smokers, smokers	Non-smokers	Binary
Alcohol consumption	Drinking status: non-drinkers, drinkers	Non-drinkers	Binary
Diabetes	Self-reported diagnosis of diabetes	Non-diabetic patients	Binary
Socioeconomic status	Adjusted social economic score categorized into tertile, created based on variables on housing quality, water supply and sanitation in a principal component analysis (PCA).	Low	Categorical
Other factors associated with the outcomes			
Index patients			
Sex	Male/female	Male	Binary
Employment status	Whether someone works outside versus staying at home.	Work at home	Binary
Household contacts			
Sex	Male/female	Male	Binary
Age groups (children HHC)	0–4;<br>5–9;<br>10–15	0–4	Categorical
Age groups (all HHC)	0–15;<br>16–30;<br>31–45;<br>46 and older;	0–15	Categorical
HIV status	HIV status from lab test and self-report	Negative	Binary
Diabetes	Self-reported diagnosis of diabetes	Non-diabetic patients	Binary
BCG scars	Any vaccination scars observed	No BCG scars	Binary
Smoking	Smoking status: non-smokers, smokers	Non-smokers	Binary
Alcohol consumption	Drinking status: non-drinkers, drinkers	Non-drinkers	Binary
BMI category	We classified adults ≥20 y old as underweight (BMI <18.5 kg/m^2), normal weight (BMI 18.5–<25 kg/m^2), and overweight (BMI ≥25 kg/m^2). For children and adolescents <20 y old, we used WHO age- and gender-specific BMI z-scores tables to classify those with BMI z-score <–2 as underweight and those with z-score >2 as overweight.	Normal weight	Categorical

Appendix Table 2. Exposure and confounder distributions between household contacts (HHCs) included and excluded from the analysis

Variables	Excluded HHCs, n = 597	Included HHCs, n = 3699	Odds ratio (OR)	P value for OR	P value for χ^2
Symptoms status					0.968
Symptomatic	578 (96.8%)	3586 (96.9%)	Ref	Ref	
Asymptomatic	19 (3.18%)	113 (3.05%)	1.04 (0.64, 1.71)	0.87	
Age (years)					0.631
16–30	328 (54.9%)	2135 (57.7%)	Ref	Ref	
31–45	154 (25.8%)	910 (24.6%)	1.10 (0.90–1.35)	0.36	
46–60	62 (10.4%)	348 (9.41%)	1.16 (0.86–1.56)	0.32	
61 and older	53 (8.88%)	306 (8.27%)	1.13 (0.82–1.54)	0.45	
HIV status					1
Negative	541 (97.5%)	3603 (97.4%)	Ref	Ref	
Positive	14 (2.52%)	96 (2.60%)	0.97 (0.55–1.71)	0.92	
Smoking status					0.803
Non-smoker	502 (96.9%)	3596 (97.2%)	Ref	Ref	
Smoker	16 (3.09%)	103 (2.78%)	1.11 (0.65–1.90)	0.70	
Socioeconomic status					0.037
Low	205 (43.2%)	1376 (37.2%)	Ref	Ref	
Medium	198 (41.8%)	1690 (45.7%)	0.79 (0.64–0.97)	0.03	
High	71 (15.0%)	633 (17.1%)	0.75 (0.57–1.00)	0.05	
Alcohol consumption					0.608
Non-drinker	248 (59.0%)	2131 (57.6%)	Ref	Ref	
Drinker	172 (41.0%)	1568 (42.4%)	0.94 (0.77–1.16)	0.57	
Diabetes status					0.567
No	537 (95.9%)	3523 (95.2%)	Ref	Ref	
Yes	23 (4.11%)	176 (4.76%)	0.86 (0.55–1.34)	0.50	
Baseline infection					0.035
No	298 (72.5%)	2858 (77.3%)	Ref	Ref	
Yes	113 (27.5%)	841 (22.7%)	1.29 (1.02–1.62)	0.03	

Appendix Table 3. Characteristics of tuberculosis index patients by symptom status. Values are n (%)

Variable	Asymptomatic, n = 113	Symptomatic, n = 2996	Odds ratio (95% CI)	p value for OR	p value for χ^2
Age (years; n = 3109):					0.579
16–30	73 (64.6)	1758 (58.7)	Ref	Ref	
31–45	19 (16.8)	636 (21.2)	1.39 (0.85–2.39)	0.21	
46–60	11 (9.7)	345 (11.5)	1.30 (0.71–2.62)	0.42	
61 and older	10 (8.9)	257 (8.6)	1.07 (0.57–2.22)	0.85	
Sex (n = 3109):					0.697
Male	68 (60.2)	1734 (57.9)	Ref	Ref	
Female	45 (39.8)	1262 (42.1)	1.10 (0.75–1.62)	0.63	
HIV status (n = 3076):					1.000
Negative	110 (97.3)	2872 (96.9)	Ref	Ref	
Positive	3 (2.7)	91 (3.1)	1.16 (0.43–4.78)	0.80	
Diabetes (n = 3083):					0.443
No	109 (96.5)	2801 (94.3)	Ref	Ref	
Yes	4 (3.5)	169 (5.7)	1.64 (0.68–5.41)	0.34	
Smoking status (n = 3055):					0.348
Non-smoker	108 (96.4)	2873 (97.6)	Ref	Ref	
Smoker	4 (3.6)	70 (2.4)	0.66 (0.27–2.19)	0.42	
Alcohol consumption status (n = 2994):					0.256
Non-drinker	67 (64.4)	1686 (58.3)	Ref	Ref	
Drinker	37 (35.6)	1204 (41.7)	1.29 (0.87–1.96)	0.22	
Employment status (n = 3093):					0.686
Stay at Home	70 (63.1)	1950 (65.4)	Ref	Ref	
Work Outside	41 (36.9)	1032 (34.6)	0.90 (0.61–1.35)	0.61	
Socioeconomic status (n = 3027):					0.364
Low	33 (31.1)	1007 (34.5)	Ref	Ref	
Medium	36 (34.0)	1080 (37.0)	0.98 (0.61–1.59)	0.95	
High	37 (34.9)	834 (28.6)	0.74 (0.46–1.19)	0.21	
Smear results (n = 3094):					**<0.001***
Negative	57 (52.8)	782 (26.2)	Ref	Ref	
Positive	51 (47.2)	2204 (73.8)	3.15 (2.14–4.65)	<0.001	

* p<0.05

Appendix Table 4. Characteristics of all household contacts by symptom status of their index patients. Values are n (%)

Variable	Total (n = 12230)	Symptomatic (n = 11758)	Asymptomatic (n = 472)
Age (years; n = 12230):			
0–15	4296 (35.1)	4164 (35.4)	132 (28.0)
16–30	3340 (27.3)	3239 (27.5)	101 (21.4)
31–45	2176 (17.8)	2104 (17.9)	72 (15.3)
46–60	1585 (13.0)	1520 (12.9)	65 (13.8)
61 and older	833 (6.8)	731 (6.2)	102 (21.6)
Sex (n = 12230):			
Male	5484 (44.8)	5219 (44.4)	265 (56.1)
Female	6746 (55.2)	6539 (55.6)	207 (43.9)
HIV status (n = 12091):			
Negative	12045 (99.6)	11581 (99.6)	464 (100)
Positive	46 (0.4)	46 (0.4)	0 (0)
BCG scar (n = 12228):			
No	1687 (13.8)	1599 (13.6)	88 (18.6)
Yes	10541 (86.2)	10157 (86.4)	384 (81.4)
Diabetes (n = 12141):			
No	11930 (98.3)	11467 (98.2)	463 (98.7)
Yes	211 (1.7)	205 (1.8)	6 (1.3)
Smoking (n = 12103):			
Non-smoker	11374 (94.0)	10941 (94.0)	433 (92.9)
Smoker	729 (6.0)	696 (6.0)	33 (7.1)
Alcohol consumption (n = 11991):			
Non-drinker	8911 (74.3)	8548 (74.1)	363 (78.9)
Drinker	3080 (25.7)	2983 (25.9)	97 (21.1)
BMI category (n = 12119):			
Normal	7010 (57.8)	6748 (57.9)	262 (55.9)
Underweight	212 (1.8)	200 (1.7)	12 (2.6)
Overweight	4897 (40.4)	4703 (40.4)	194 (41.5)

Appendix Table 5. Risk of *Mycobacterium tuberculosis* infection at baseline among all household contacts of tuberculosis index patients by symptom status

Symptom status of index patients	Numbers of HHC, N	Baseline infection, N (%)	Crude prevalence ratio (95% CI)	Model A		Model B		Model C	
				Prevalence ratio (95% CI)	P value	Prevalence ratio (95% CI)	P value	Prevalence ratio (95% CI)	P value
Symptomatic	9,214	3,430 (37.23)	Ref	Ref		Ref		Ref	
Asymptomatic	359	116 (32.31)	0.87 (0.75–1.01)	0.94 (0.78–1.13)	0.51	0.94 (0.79–1.11)	0.47	0.94 (0.79–1.11)	0.45

Model A: univariable model
Model B: adjusted for the following characteristics of index patients: age, sex, HIV status, smoking status, alcohol consumption status socioeconomic status, employment status, and diabetes; and the following characteristics of household contacts: age, sex, HIV status, smoking status, alcohol consumption status, diabetes, BCG vaccination, and BMI category.
Model C: Model B without adjusted for employment status of index patients and HIV status, BCG scar, smoking, and alcohol consumption of household contacts.

Appendix Table 6. Risk of *Mycobacterium tuberculosis* infection at baseline among household contacts (HHCs) of tuberculosis index patients by symptom status using multiple imputation

Population	Symptom status of index patients	Model A		Model B		Model C	
		Prevalence ratio (95% CI)	P value	Prevalence ratio (95% CI)	P value	Prevalence ratio (95% CI)	P value
HHCs under the age of 15	Symptomatic	Ref		Ref		Ref	
	Asymptomatic	0.56 (0.34–0.92)	0.02	0.58 (0.35–0.95)	0.03	0.57 (0.35–0.94)	0.03
All HHCs	Symptomatic	Ref		Ref		Ref	
	Asymptomatic	0.88 (0.73–1.05)	0.09	0.88 (0.74–1.04)	0.13	0.88 (0.74–1.04)	0.13

Model A: univariable model
Model B: adjusted for the following characteristics of index patients: age, sex, HIV status, smoking status, alcohol consumption status socioeconomic status, employment status, and diabetes; and the following characteristics of household contacts: age, sex, HIV status, smoking status, alcohol consumption status, diabetes, BCG vaccination, and BMI category.
Model C: Model B without adjusted for employment status of index patients and HIV status, BCG scar, smoking, and alcohol consumption of household contacts.

Appendix Table 7. The hazard of Mycobacterium tuberculosis infection at 6 mo of follow-up among initially uninfected all household contacts of tuberculosis patients by symptom status.

Symptom status of index patients	Number of HHC	Incident infection (n, %)	Model A		Model B		Model C	
			Hazard ratio (95% CI)	P value	Hazard ratio (95% CI)	P value	Hazard ratio (95% CI)	P value
Symptomatic	4,547	1,123 (24.70)	Ref		Ref		Ref	
Asymptomatic	190	38 (20.00)	0.80 (0.53–1.23)	0.31	0.78 (0.50–1.20)	0.26	0.78 (0.50–1.20)	0.26

Model A: univariable model
Model B: adjusted for the following characteristics of index patients: age, sex, HIV status, smoking status, alcohol consumption status socioeconomic status, employment status, and diabetes; and the following characteristics of household contacts: age, sex, HIV status, smoking status, alcohol consumption status, diabetes, BCG vaccination, BMI category.
Model C: Model B without adjusted for: employment status of index patients and HIV status, diabetes, smoking, alcohol consumption, and BMI category of household contact

Appendix Table 8. The hazard of *Mycobacterium tuberculosis* infection at 12-mo of follow-up among initially uninfected household contacts of tuberculosis patients under the age of 15 by symptom status

			Model A		Model B		Model C	
Symptom status of index patients	Number of HHC	Incident infection (n, %)	Hazard ratio (95% CI)	P value	Hazard ratio (95% CI)	P value	Hazard ratio (95% CI)	P value
Symptomatic	2,204	465 (21.10)	Ref		Ref		Ref	
Asymptomatic	68	11 (16.18)	0.75 (0.37–1.53)	0.43	0.73 (0.35–1.50)	0.38	0.73 (0.35–1.50)	0.39

Model A: univariable model
Model B: adjusted for the following characteristics of index patients: age, sex, HIV status, smoking status, alcohol consumption status socioeconomic status, employment status, and diabetes; and the following characteristics of household contacts: age, sex, HIV status, alcohol consumption status, BCG vaccination, and BMI category. (Diabetes and smoking status of household contacts excluded due to sparse data in some of its categories, which led to unstable hazard ratio estimates and non-estimable coefficients (NA) in the Cox model)
Model C: Model B without adjusted for sex and alcohol consumption status of household contacts.

Appendix Table 9. The hazard of *Mycobacterium tuberculosis* infection at 12-mo of follow-up among initially uninfected all household contacts of tuberculosis patients by symptom status

			Model A		Model B		Model C	
Symptom status of index patients	Number of HHC	Incident infection (n, %)	Hazard ratio (95% CI)	P value	Hazard ratio (95% CI)	P value	Hazard ratio (95% CI)	P value
Symptomatic	4,547	1,532 (33.69)	Ref		Ref		Ref	
Asymptomatic	190	50 (26.32)	0.82 (0.57–1.18)	0.29	0.80 (0.55–1.17)	0.25	0.80 (0.54–1.16)	0.24

Model A: univariable model
Model B: adjusted for the following characteristics of index patients: age, sex, HIV status, smoking status, alcohol consumption status socioeconomic status, employment status, and diabetes; and the following characteristics of household contacts: age, sex, HIV status, smoking status, alcohol consumption status, diabetes, BCG vaccination, BMI category.
Model C: Model B without adjusted for: employment status of index patients, and HIV status, smoking, and alcohol consumption of household contact

Appendix Table 10. The hazard of *Mycobacterium tuberculosis* infection and disease among initially uninfected household contacts (HHCs) of tuberculosis patients by symptom status using multiple imputation

Outcome and population	Symptom status of index patients	Model A		Model B		Model C	
		Hazard ratio (95% CI)	P value	Hazard ratio (95% CI)	P value	Hazard ratio (95% CI)	P value
Incident infection at 6 mo of follow-up							
HHCs under the age of 15	Symptomatic	Ref		Ref		Ref	
	Asymptomatic	0.67 (0.31–1.43)	0.29	0.65 (0.29–1.44)	0.29	0.65 (0.29–1.44)	0.29
All HHCs	Symptomatic	Ref		Ref		Ref	
	Asymptomatic	0.88 (0.60–1.28)	0.49	0.85 (0.58–1.26)	0.42	0.86 (0.58–1.26)	0.44
Incident infection at 12 mo of follow-up							
HHCs under the age of 15	Symptomatic	Ref		Ref		Ref	
	Asymptomatic	0.77 (0.41–1.44)	0.41	0.74 (0.39–1.41)	0.36	0.74 (0.39–1.41)	0.36
All HHCs	Symptomatic	Ref		Ref		Ref	
	Asymptomatic	0.87 (0.63–1.21)	0.42	0.86 (0.61–1.20)	0.37	0.86 (0.61–1.20)	0.49
Incident disease at 12 mo of follow-up							
All HHCs	Symptomatic	Ref		Ref		Ref	
	Asymptomatic	0.55 (0.26–1.17)	0.12	0.61 (0.29–1.28)	0.19	0.62 (0.29–1.30)	0.20

Model A: univariable model
Model B: adjusted for the following characteristics of index patients: age, sex, HIV status, smoking status, alcohol consumption status socioeconomic status, employment status, and diabetes; and the following characteristics of household contacts: age, sex, HIV status, smoking status, alcohol consumption status, diabetes, BCG vaccination, BMI category.
Model C:
For the outcome of incident infection at 6 mo of follow-up among all HHCs: Model B without adjusted for: employment status of index patients and HIV status, diabetes, smoking, alcohol consumption, and BMI category of household contact
For the outcome of incident infection at 12 mo of follow-up among all HHCs: Model B without adjusted for: employment status of index patients, and HIV status, smoking, and alcohol consumption of household contact
For the outcome of incident infection at 6 mo of follow-up among HHCs under the age of 15: Model B without adjusted for: sex and alcohol consumption of household contact
For the outcome of incident infection at 12 mo of follow-up among HHCs under the age of 15: Model B without adjusted for: sex and alcohol consumption of household contact
For the outcome of incident disease among all HHCs: Model B without adjusted for employment status of index patients, and sex, smoking status, and alcohol consumption of household contact

Appendix Table 11. The hazard of incident *Mycobacterium tuberculosis* infection among household contacts under the age of 15 by symptom pattern of index patients

Symptom pattern of index patients	Number of HHC	Incident TB infection (N, %)	Model A		Model B		Model C	
			Hazard ratio (95% CI)	P value	Hazard ratio (95% CI)	P value	Hazard ratio (95% CI)	P value
Asymptomatic	68	7 (10.29)	Ref		Ref		Ref	
Cough only	165	26 (15.76)	1.78 (0.68–4.66)	0.24	1.84 (0.65–5.21)	0.25	1.84 (0.69–4.94)	0.23
Non-cough symptoms only	158	18 (11.39)	1.25 (0.46–3.40)	0.66	1.31 (0.44–3.88)	0.62	1.27 (0.46–3.55)	0.65
Cough and any non-cough symptoms	1842	273 (14.82)	1.61 (0.68–3.78)	0.28	1.66 (0.66–4.18)	0.28	1.59 (0.66–3.81)	0.30

Model A: univariable model
Model B: adjusted for the following characteristics of index patients: age, sex, HIV status, smoking status, alcohol consumption status socioeconomic status, employment status, and diabetes; and the following characteristics of household contacts: age, sex, HIV status, smoking status, alcohol consumption status, diabetes, BCG vaccination, and BMI category.
Model C: Model B without adjusted for employment status and sex of index patients, and sex, smoking status, and alcohol consumption of household contact
* Non-cough symptoms include fever, weight loss and night sweat.

.

Appendix Table 12. Univariate analysis of the association between characteristics of asymptomatic index patients and risk of *Mycobacterium tuberculosis* infection at baseline of all household contacts

Characteristics of asymptomatic index patients	Uninfected, n = 297	Infected, n = 137	Prevalence ratio (95%CI)	P value for PR	P value for χ^2
Age (n = 434):					0.020*
16–30	151 (50.8%)	80 (58.4%)	Ref	Ref	
31–45	33 (11.1%)	18 (13.1%)	1.01 (0.66, 1.56)	0.96	
46–60	37 (12.5%)	22 (16.1%)	1.09 (0.64, 1.86)	0.75	
61 and older	76 (25.6%)	17 (12.4%)	0.62 (0.36, 1.08)	0.09	
Sex (n = 434):					0.031*
Male	228 (76.8%)	91 (66.4%)	Ref	Ref	
Female	69 (23.2%)	46 (33.6%)	1.26 (0.90, 1.76)	0.18	
HIV status (n = 434):					0.004*
Negative	234 (78.8%)	124 (90.5%)	Ref	Ref	
Positive	63 (21.2%)	13 (9.49%)	0.54 (0.32, 0.92)	**0.02***	
Smoking status (n = 433):					0.856
Non-smoker	278 (93.9%)	130 (94.9%)	Ref	Ref	
Smoker	18 (6.08%)	7 (5.11%)	0.69 (0.34, 1.39)	0.29	
SES (n = 408):					0.060
Low	78 (27.8%)	35 (27.6%)	Ref	Ref	
Medium	157 (55.9%)	59 (46.5%)	1.04 (0.67, 1.63)	0.85	
High	46 (16.4%)	33 (26.0%)	1.36 (0.84, 2.20)	0.21	
Employment status (n = 431):					0.985
Stay at Home	210 (71.4%)	97 (70.8%)	Ref	Ref	
Work Outside	84 (28.6%)	40 (29.2%)	0.80 (0.56, 1.15)	0.23	
Alcohol consumption (n = 392):					0.122
Drinker	76 (28.7%)	47 (37.0%)	Ref	Ref	
Non-drinker	189 (71.3%)	80 (63.0%)	0.82 (0.57, 1.17)	0.27	
Diabetes (n = 434):					0.079
No	293 (98.7%)	131 (95.6%)	Ref	Ref	
Yes	4 (1.35%)	6 (4.38%)	1.56 (0.65, 3.73)	0.32	

*: $p < 0.05$

Appendix Table 13. Tuberculosis infection and disease among household contacts (HHCs) of tuberculosis patients by symptom status

	Baseline infection among child HHCs		Incident infection at 6 mo among child HHCs		Incident infection at 12 mo among child HHCs		Incident disease at 12 mo among all HHCs	
Index patient coughing duration (days)	Adjusted prevalence ratio (95% CI)	P value	Adjusted hazard ratio (95% CI)	P value	Adjusted hazard ratio (95% CI)	P value	Adjusted hazard ratio (95% CI)	P value
Asymptomatic	Ref		Ref		Ref		Ref	
0–13	1.28 (0.74–2.19)	0.37	1.34 (0.52–3.40)	0.54	1.16 (0.54–2.51)	0.70	0.93 (0.42–2.06)	0.85
14–28	1.56 (0.93–2.63)	0.09	1.62 (0.66–3.99)	0.29	1.42 (0.68–2.96)	0.36	1.23 (0.58–2.63)	0.59
29–55	1.73 (1.03–2.92)	0.04	1.76 (0.71–4.38)	0.22	1.43 (0.68–3.01)	0.35	1.48 (0.69–3.19)	0.31
≥56	1.76 (1.04–2.98)	0.04	1.61 (0.64–4.03)	0.31	1.44 (0.68–3.05)	0.35	1.31 (0.61–2.85)	0.49

Model adjusted for the following characteristics of index patients: age, sex, HIV status, smoking status, alcohol consumption status socioeconomic status, employment status, and diabetes; and the following characteristics of household contacts: age, sex, HIV status, smoking status, alcohol consumption status, diabetes, BCG vaccination, BMI category

Appendix Table 14. Univariate analysis of the association between characteristics of asymptomatic index patients and risk of *Mycobacterium tuberculosis* disease of all household contacts

Characteristics of asymptomatic index patients	Non-patients, n = 76	Patients, n = 9	Odds ratio (95% CI)	P value for OR	P value for χ^2
Age (n = 85):					0.456
16–30	50 (65.8%)	6 (66.7%)	Ref.	Ref.	
31–45	15 (19.7%)	1 (11.1%)	4.62 (0.69–31.1)	0.12	
46–60	6 (7.89%)	2 (22.2%)	1.86 (0.2–17.3)	0.59	
61 and older	5 (6.58%)	0 (0.00%)	2.45 (0.37–16.1)	0.35	
Sex (n = 85):					0.711
Male	50 (65.8%)	7 (77.8%)	Ref.	Ref.	
Female	26 (34.2%)	2 (22.2%)	0.61 (0.12, 3.18)	0.56	
HIV status (n = 85):					0.366
Negative	73 (96.1%)	8 (88.9%)	Ref.	Ref.	
Positive	3 (3.95%)	1 (11.1%)	2.46 (0.42, 14.3)	0.32	
Smoking status (n = 84):					1.000
Non-smoker	70 (93.3%)	9 (100%)	Ref.	Ref.	
Smoker	5 (6.67%)	0 (0.00%)	-	-	
SES (n = 82):					0.068
Low	37 (50.7%)	3 (33.3%)	Ref.	Ref.	
Medium	31 (42.5%)	3 (33.3%)	3.64 (0.41–32.7)	0.25	
High	5 (6.85%)	3 (33.3%)	2.55 (0.22–29.2)	0.45	
Employment status (n = 83):					0.170
Stay at Home	38 (51.4%)	7 (77.8%)	Ref.	Ref.	
Work Outside	36 (48.6%)	2 (22.2%)	0.54 (0.06–4.49)	0.57	
Alcohol consumption (n = 75):					0.462
Drinker	24 (35.8%)	4 (50.0%)	Ref.	Ref.	
Non-drinker	43 (64.2%)	4 (50.0%)	0.90 (0.18–4.57)	0.90	
Diabetes (n = 85):					1.000
No	75 (98.7%)	9 (100%)	Ref.	Ref.	
Yes	1 (1.32%)	0 (0.00%)	-	-	
